# A technical review of the history, development and performance of the anaesthetic conserving device “AnaConDa” for delivering volatile anaesthetic in intensive and post-operative critical care

**DOI:** 10.1007/s10877-017-0097-9

**Published:** 2018-01-31

**Authors:** Ron Farrell, Glen Oomen, Pauric Carey

**Affiliations:** 1 Sedana Medical, The Village Center, Two Mile House, Naas, Co. Kildare Ireland; 2 Sedana Medical, Unit 306, 397 King Street West, Dundas, ON L9H 1W9 Canada

**Keywords:** Sedation, Critical care, Isoflurane, Sevoflurane, Inhaled anaesthetic, Anaesthetic conserving device, AnaConDa, Mechanical ventilation

## Abstract

There is a shift in critical care to adopt volatile anaesthetics as sedatives for certain patients using mechanical ventilation. Accompanying this shift is a growing body of literature describing the advantages or disadvantages of using isoflurane or sevoflurane for long term sedation. This practise requires a cost effective, efficient and safe means to deliver these drugs that can simultaneously operate with modern critical care ventilators and ventilation protocols while protecting the care environment and care workers from excessive exposure to the drugs. The anaesthetic conserving device (“AnaConDa”, Sedana Medical) is one device that delivers a safe sedative dose of either isoflurane or sevoflurane to a patient using existing critical care ventilators, common syringe pumps and gas monitors. The device is essentially a small disposable anaesthetic vaporizer and HME filter combined into one airway component. Similar to an HME filter, the device reflects moisture back to the patient, but also reflects 90% of the anaesthetic by adsorbing and releasing the drug using a proprietary carbon filament reflecting medium. This reflection reduces the total amount of anaesthetic needed, reducing that which is exhausted or scavenged upon exhalation. It can be used for 24 h of sedation, and fits into current critical care ventilator circuits almost without modifications. This article will describe the physical characteristics of the device, how it works, its development history and the performance parameters under which it can be used.

## Introduction

The anaesthetic conserving device, “AnaConDa” (Sedana Medical, Danderyd Sweden) is a simple, disposable, class IIa device that allows the inhaled anaesthetics isoflurane and sevoflurane to be safely and efficiently vaporized and delivered utilizing any non-rebreathing mechanical ventilator. While initially intended to administer isoflurane and sevoflurane in the operating room for general anesthesia, it now is most frequently used to sedate mechanically ventilated patients in the intensive care unit. To date, over 2300,000 AnaConDa’s have been used in hospitals globally. There is a growing body of literature debating the clinical use of inhaled anaesthetics as sedatives. While this article will cite some of that literature, the focus will be on reviewing the technical aspects of the device, the background leading to its development, and its in vitro performance characteristics.

**Fig. 1:**
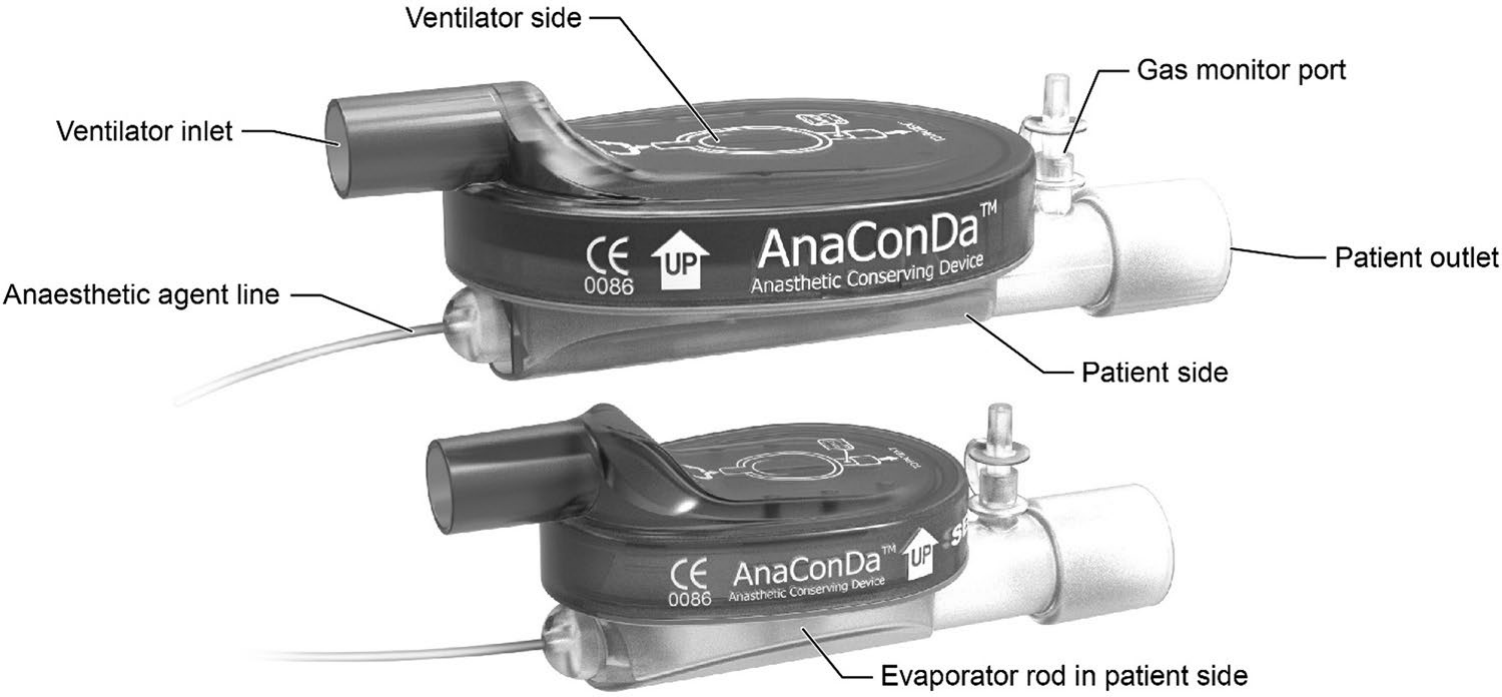
The 100 mL AnaConDa (top) in comparison to the 50 mL AnaConDa (bottom), showing the basic external features common to both devices

The AnaConDa device (Fig. 1) fits into a ventilator circuit, replacing the heat and moisture exchanger (HME) filter between the Y-piece and endotracheal tube or T-piece. The AnaConDa carries out all the vital requirements of an efficient and safe anaesthetic delivery system while being completely passive. It is not powered in any way. On its own, it has no measurement capability and no means for providing a readout. Like a HME, the device has a patient side and a ventilator side separated by a filter. The filter is comprised of an electrostatic polypropylene anti-bacterial/anti-viral layer to protect the ventilator from contamination, and a thicker carbon felt layer for adsorbing and reflecting organic vapors and moisture. Mounted within the patient side of the device is a porous polypropylene evaporator rod. The evaporator rod is fed liquid isoflurane or sevoflurane through a 2 m polyethylene external agent line from a modified 50 mL syringe mounted in a standard syringe pump. The AnaConDa cannot be used to administer desflurane because it boils at room temperature.

During the breathing cycle, the gas coming from the inspiratory limb picks up the vapor from the evaporator rod, and the resulting mixture is inhaled by the patient. As not all anaesthetic vapor will be absorbed by the patient, the exhaled gas will still contain vapor. This vapour in the exhaled breath will be adsorbed in the carbon filter. During the next inspiration, 90% of this adsorbed amount will be released again, or “reflected” towards the patient. Optimally, this anaesthetic reflection is approximately 90% efficient, with the remaining 10% reaching the exhalation limb where it should be scavenged at the ventilator exhaust. Some degree of inefficiency is required to allow the inhaled anaesthetic concentration to be decreased if need be. A gas monitor connected to a port on the patient side of the device measures the inspired and expired CO_2_, and end-tidal concentration of the drug. The clinician-user monitors agent concentration in the exhaled breath (F_ET_) in conjunction with the patient sedation depth, as assessed by RASS or other suitable methods. Together, these inform the infusion rate of the pump to meet the recommended target F_ET_ concentration for the desired sedation level. The device is currently available with two different internal volumes, 100 and 50 mL, each suiting patient over a different range of tidal volumes. Thus, an anaesthetic gas monitor, a ventilator and syringe pump can be used with this device to deliver isoflurane or sevoflurane at safe sedative doses to a patient in critical care. The AnaConDa device is distributed as a clean, non-sterile disposable device intended for single use. It should be replaced after 24 h of use.

## History and development

The idea of the AnaConDa was conceived in the mid 1990s by Louis Gibeck AB, the original developer of the disposable HME. It was postulated that, if moisture and heat could be trapped by a filter and reflected back to the patient, perhaps so too could volatile anaesthetic. Such a device would utilize the high fresh gas flow (FGF) of open circuit anaesthesia systems, but minimize the waste typical of them by returning exhaled volatile anaesthetic to the patient instead of exhausting that agent into the environment. In addition, liquid anaesthetic agent would be fed by a conventional syringe and pump into an evaporator within the device, functioning as miniature vaporizer. The envisioned device would reliably reflect anaesthetic, heat and moisture, provided that a suitable reflection medium could be found. Over 200 materials were tested for suitability and reflective performance during development.

The first version of this anaesthetic conserving device (ACD) was named the Alfa-reflector. Produced in a limited number for trial, it received its CE mark in October 1999. The use of the ACD during anesthesia, as conceived by its inventor Hans Lambert, was first documented using isoflurane in 2001 [1] The prototype allowed a 40% reduction in the consumption of isoflurane through reflection compared to contemporary practise with high FGF, and more than 55% reduction in the amount of agent exhausted and lost to the atmosphere or scavenging. Using sevoflurane and an improved version of the device, they then compared its performance to the use of vaporizers during low flow anesthesia, finding similar efficiency between both systems [2]. This improved “Beta-Reflector”, nearly identical to the current 100 mL device, received its CE mark in 2003, and was made available by Hudson RCI under the name “AnaConDa”, shortened from anaesthetic conserving device. Tempia et al. subsequently compared the ACD to conventional circle systems over a range of FGF, and also found it to be as efficient as then state-of-art low-flow anaesthesia with conventional vaporizer systems. They also noted that by simply stopping the pump and completely disconnecting the ACD from the circuit, there was relatively rapid washout of the drug [3].

In 2004, Sackey et al. published findings from the first trial using the AnaConDa for long term (> 12 h) sedation in the ICU, comparing its use and the quality of sedation to intravenously administered midazolam. Their findings suggested that using isoflurane administered by the ACD resulted in notably shorter wake-up times than their midazolam group. Despite having little practical experience with inhalation agents, they noted that the ICU nursing staff were able to safely titrate doses and monitor sedation [4].

In 2005 Sedana Medical AB acquired the AnaConDa technology and continued development of it. Based on clinician feedback regarding the range of minute volumes in which the 100 mL device was clinically useful, it was thought that a much smaller version of the device was needed. This feedback suggested that the dead space of the 100 mL AnaConDa was too large for patients (< 50 kg) with small tidal volumes, or for those with impaired or limited lung function in which even small increases in dead space ventilation was detrimental. With the existing 100 mL device, normocapnia was difficult to achieve at tidal volumes below 350 mL. A 50 mL version of the AnaConDa was developed and subsequently released in 2017 to address this need. With design refinements, even at half the size, the 50 mL AnaConDa has reflection and resistance performance very similar to the 100 mL device. The 50 mL AnaConDa is suitable for use with tidal volumes as low as 200 mL.

## Detailed description of the AnaConDa

**Fig. 2:**
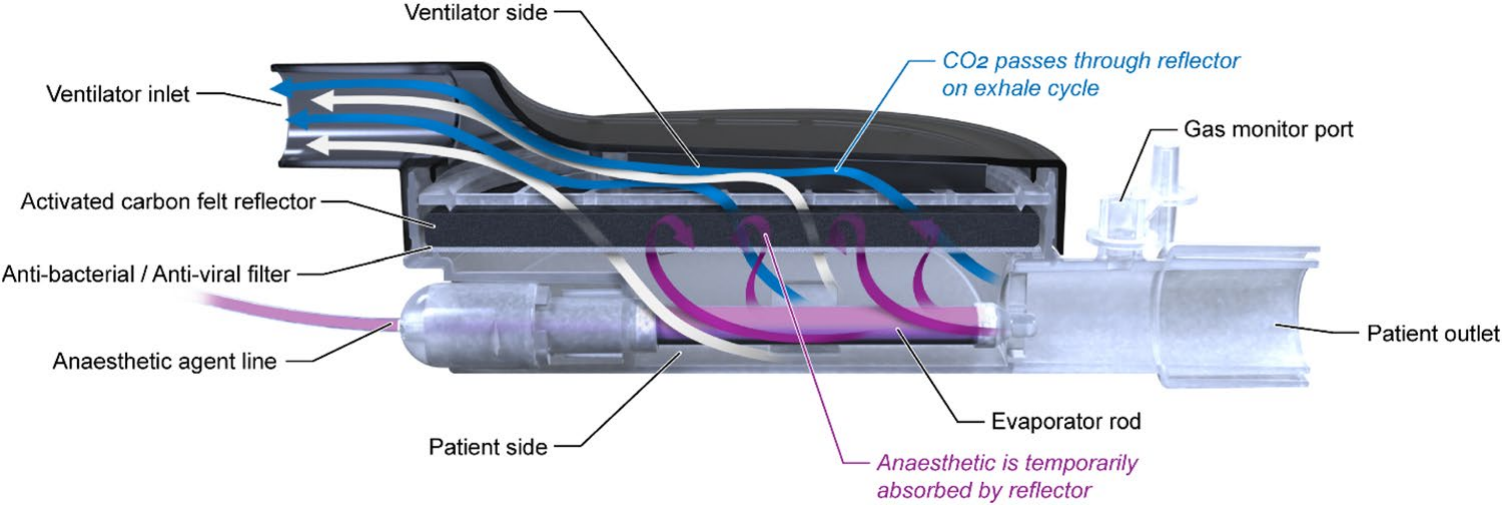
Cross section of the AnaConDa device showing filter media, evaporator rod and schematic airflow during exhalation

The AnaConDa, like a traditional HME, has two halves—a patient side and a ventilator side—separated by filters. The AnaConDa, Fig. 2, has an inlet on the ventilator side enabling it to be connected to a standard Y-piece or the inspiratory limb of the ventilator circuit. The patient side of the device has an outlet to connect directly to an endotracheal tube or flex tube and suction T-piece. The patient outlet has a gas sampling port and cap with standard luer thread onto which a gas sampling line and monitor are connected when in use.

The housing is made of two molded polyethylene halves, one clear for the patient side, the other black for the ventilator side and assembled by snap fit and glue. Assembled, the two halves sandwich the filters and support grid between them. The housing has a unique oval shape, with the ventilator inlet and patient outlet oriented parallel to the surface of the filter medium. This orientation is designed to maintain organized laminar air flow within the device, moving air over the inside of the housing and directing it over the entire surface area of the filter, making optimal use of it. Both 50 and 100 mL AnaConDa share this form.

The device comes with a red polypropylene cap covering the patient outlet. This cap, obviously, is removed prior to use and kept on hand. During disposal, the red cap is replaced to prevent anaesthetic vapor leaking into the environment.

The filter of the AnaConDa consists of two layers. On the patient side of the device is a white anti-bacterial and anti-viral filter, typical of HME filters. This layer primarily protects the carbon filter and ventilator components from contamination. The AnaConDa differs from HME filters in having a second layer comprised of a 3–4 mm thick pad of activated carbon felt. This proprietary carbon felt is capable of adsorbing and releasing isoflurane or sevoflurane vapor. These vapors are released again upon inhalation, returning the drug to the patient and generating the reflection effect of the device.

The evaporator rod is mounted within the patient side of the housing. The evaporator is made of a porous polypropylene extruded rod. It is pressed into a mount securing it within the housing and connecting it to the polyethylene agent line that supplies it with liquid isoflurane or sevoflurane from the syringe.

The agent line has a red polypropylene adaptor on the end that screws onto a modified 50 mL syringe provided by Sedana Medical. The agent line adaptor has a small spring valve that only opens when affixed fully to the syringe. This prevents the drug from leaking backwards out of the agent line towards the ACD when the syringe is disconnected from the device for refilling. Instead of the ubiquitous luer thread, the agent line adaptor and modified syringe share a thread pitch and diameter unique among medical devices so that they are exclusively “keyed” to each other. A common syringe cannot be threaded onto the AnaConDa; similarly, the modified syringe cannot be threaded onto the luer adaptors of other medical devices. This is a safeguard against human error, preventing drugs other than isoflurane or sevoflurane from being injected into the AnaConDa, and isoflurane or sevoflurane from being accidentally injected intravenously.

It is important to note that both isoflurane and sevoflurane are solvents and plasticizers capable of degrading plastics. All plastic components in the AnaConDa are made from stable polypropylene and polyethylene, or similarly stable long chain plastics such as polyoxymethylene (POM) to resist this degradation. Stainless steel springs and pins are used on the agent line adaptor one-way valve. The plasticizing characteristics of the drugs are important to consider when selecting additional ventilator components for use with volatiles, such as Y-pieces or similar adaptors. For example, hard, clear polycarbonate components can be degraded by the exhausted anaesthetic.

Sedana Medical also manufactures and distributes reusable, machined POM bottle adaptors for filling the modified syringes. These adaptors can be screwed securely onto the standard 250 mL brown glass or plastic isoflurane or sevoflurane bottles, and have a sprung stainless steel valve with matching thread for the modified syringe. Once the adaptor is screwed on to the bottle, the modified syringe can subsequently be screwed onto it, opening the spring valve and enabling fluid to be withdrawn. Inverted, a bolus of air can be injected into the bottle and the drug can be extracted carefully from it to fill the syringe with the desired dose. Care must be taken when withdrawing the anaesthetic to avoid boiling the fluid. However, any bubbles that do form can be injected back into the bottle. When the bottle is empty, the adaptor can be unscrewed and placed on the next full bottle.

## Performance

### Agent concentration

The 100 mL AnaConDa is recommended for use within tidal volumes (V_T_) ranging from 350 to 1200 mL, making it suitable for patients larger than 60 kg who have normal respiratory demands. The 50 mL AnaConDa facilitates smaller tidal volumes, from 200 to 750 mL, and thus smaller patients, down to approximately 30 kg. Both devices were validated by the manufacturer in bench-top tests according to ISO-9360 for HME and HME filters.

**Fig. 3:**
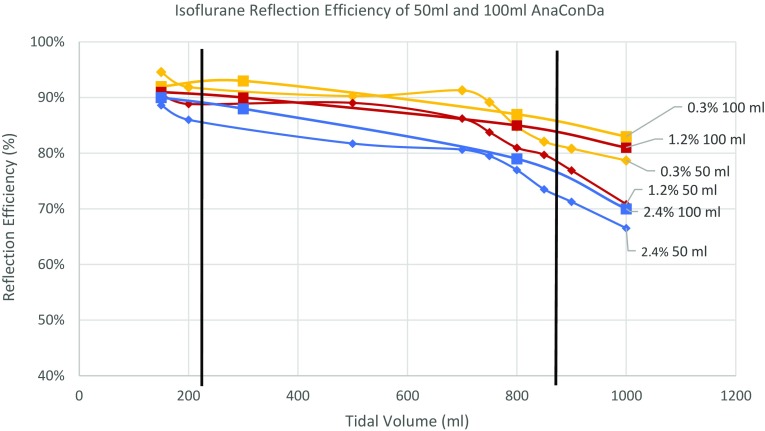
Isoflurane Reflection Efficiency of 50 and 100 mL AnaConDa at 0.3, 1.2 and 2.4% concentrations. Black bars indicate the lowest and highest tidal volume that the devices are recommended for use

Tests of reflection efficiency were performed on both devices across a range of tidal volumes, from 150 to 1000 mL, using three different drug concentrations to represent a low concentration at 0.3% F_ET_, a median concentration 1.2% F_ET_, and the relatively high 2.4% F_ET_. This is seen in Fig. 3, plotting isoflurane reflection efficiency for both devices and tidal volume at the tested drug concentrations. The 50 mL device is approximately 2% less efficient than the 100 mL device, meaning that a slightly higher infusion rate is needed when using it to provide the same drug concentration. This equates to approximately 0.2 mL per hour and is relatively insignificant. The upper limit for tidal volume of the 50 mL device was defined as the point where the reflection efficiency falls below 80% for the median drug concentration of 1.2% F_ET_. This is at approximately 850 mL.

With regards to reflection, Meiser et al. found during their bench studies that the concentration of anaesthetic agent on the evaporator or patient side of the reflection medium was always roughly ten times greater than that on the ventilator side [5]. This is consistent with the findings above. There is always a small fraction of the drug that passes through the reflector filter as waste regardless of the dose and tidal volume. Ideally, no anaesthetic would be lost through the reflection medium, however, this 10% loss does give the device a degree of steerability needed to allow the user to decrease F_ET_ relatively quickly. This loss also highlights the need for scavenging at the ventilator exhaust point. In several studies the ambient concentration of these drugs in the ICU has been reported with the use of scavenging. In each of these studies, the ambient concentrations of the drugs did not exceed the safe workplace exposure values outlined in Germany (10 ppm isoflurane), Britain (50 ppm isoflurane) or North America (2 ppm isoflurane) [6–11].

### HME performance

**Fig. 4:**
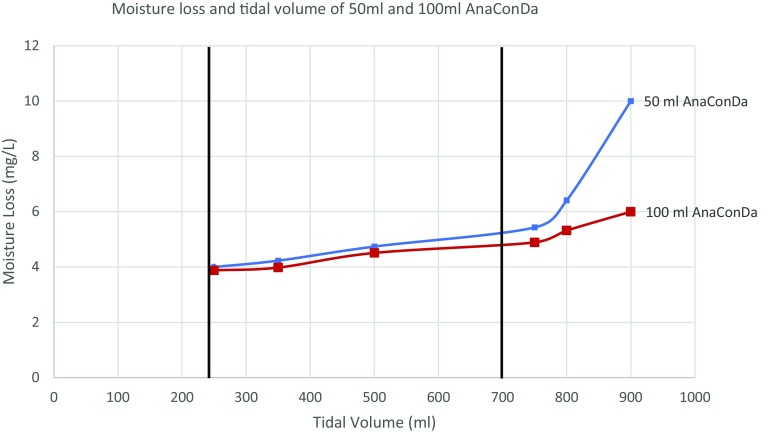
Moisture loss and tidal volume of 50 and 100 mL AnaConDa

During moisture loss testing, the simulated “lung” exhales through a heated water bath into the HME. The mass of the water bath is recorded at the beginning of the test and again after a series of “breaths” of known, consistent tidal volume. The loss is measured in mg of water lost per litre of breath. The results of testing both AnaConDa devices across a series of tidal volumes are shown in both Table 1 and Fig. 4 below. Moisture loss through the AnaConDa was also tested in comparison with the nearest analogous product, the 35 mL Humid Vent Compact Straight HME (HV-HME, Teleflex). At 500 and 750 mL tidal volumes, the moisture loss of both 100 and 50 mL AnaConDa was comparable to the HV-HME as seen in the following table. Because of this similarity in performance, the manufacturer believes that the moisture reflection efficiency of the devices is sufficient for ICU use.

**Table 1:** Comparative moisture loss at 500 and 700 mL tidal volumes between 50 and 100 mL AnaConDa, and nearest analogous HME filter

Device	Tidal volume (T_V_) (mL)	Moisture loss (mg/L)	Moisture output (mg/L H_2_O)
50 mL AnaConDa	500	5	32
100 mL AnaConDa	500	5	32
35 mL HV-HME	500	6	31
50 mL AnaConDa	750	5.5	31.5
100 mL AnaConDa	750	5	31
35 mL HV-HME	750	7	30

### Airway resistance

**Fig. 5:**
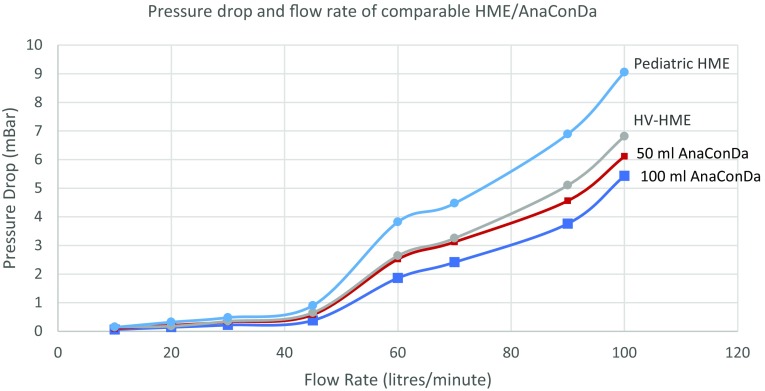
Pressure drop and flow rate of comparable HME/AnaConDa

The oval shape of the AnaConDa is designed to minimize air flow resistance through it while maximizing filter reflection. Instead of forcing air through a limited region in the middle of the filter, like in most HME’s, the shape of the AnaConDa has been designed to reduce turbulence through it and distribute the air over the entire surface area of the filter. This flow decreases the pressure differential—or “pressure drop”—across the filter. The magnitude of this pressure drop is directly related to the resistance of the device to air flowing through it. Minimizing this resistance has clinical significance because it affects the work of breathing (WOB). This is particularly critical during supportive ventilation, weaning and spontaneous breathing when patients must inhale and exhale on their own through the device [12]. Arieli et al. noted that standard HME devices reached pressure differentials of 6.0 cm H_2_O at 60 L/min peak flow rates [13], and seemed to affect the WOB much less than the endotracheal tube they were attached to. A comparison of pressure differentials between the analogous 35 mL HV-HME, a smaller (< 25 mL) pediatric HME, and both 100 and 50 mL AnaConDa can be seen in Fig. 5, indicating similar resistance between the devices. The test measured the pressure drop during 5 s intervals at increasing flow rates. While the 50 mL AnaConDa imposes more resistance than 100 mL AnaConda, the results also indicate that the pressure differential of both devices are well below Arieli’s benchmark of 5.9 mbar (6 cm H_2_O) for clinically acceptable resistance at relevant flow rates.

## Typical set-up

**Fig. 6:**
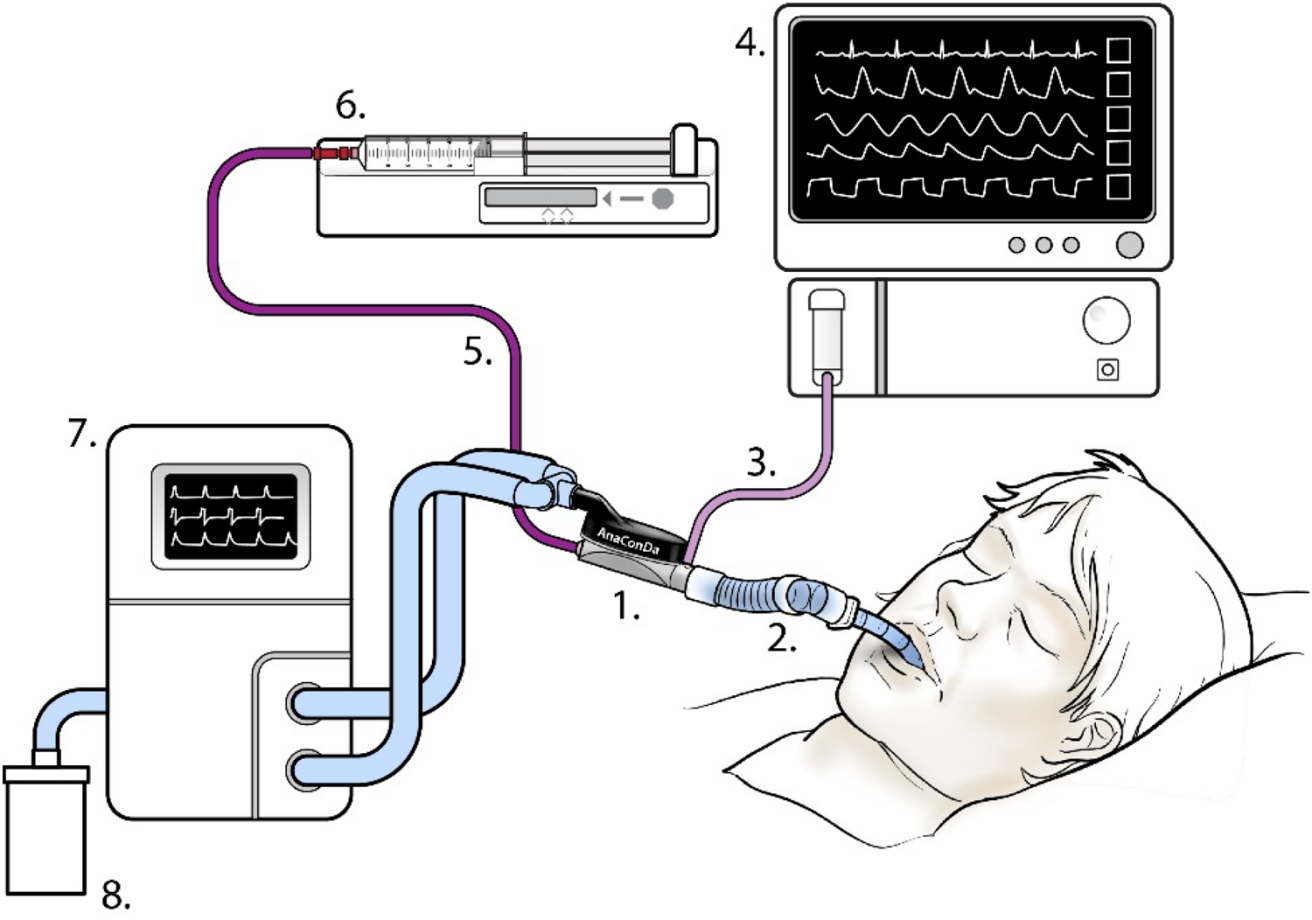
Schematic showing typical set-up with AnaConDa device

A typical set-up with the AnaConDa is seen in Fig. 6 below. The AnaConDa (1) is installed in place of the HME filter in the ventilator circuit, connected to the Y-piece and the endotracheal tube or flex-tube, (2) the AnaConDa should be oriented at an angle, tilted slightly downward towards the patient, as shown, to prevent fluid from accumulating in it. The AnaConDa gas sampling line, (3) is plugged into a gas monitor or patient monitoring unit with gas module, (4) the sedative agent line, (5) is threaded onto a full syringe placed in the syringe pump, (6) the ventilator settings, (7) have to account for the increased dead space of the AnaConDa and flex-tube or adaptor. Scavenging can be assured by affixing a scavenging canister to the exhaust port of the ventilator or other means.

Sackey documented a modified version of this set-up for use with pediatric patients, for whom the dead space of a 100 mL AnaConDa, was simply too large, resulting in hypercapnia. In this variant, his group placed the AnaConDa on the inspiratory limb before the Y-piece, using it to simply deliver drug. This obviated the reflection function of the device [14]. This set-up is outside the intended use of the device and, according to the physicians who have conducted the practice, is a solution of last resort for some patients. With such a practise, scavenging would be critical to minimize the concentration of the exhausted drug in the care environment.

## Contraindications, problems and warnings

At the time of writing, more than 300,000 clinical uses of the AnaConDa have occurred, and 62 clinician led studies have been reported on, involving 1186 patients who were exposed to the device during their treatment. Manufacturing and use of both 100 and 50 mL AnaConDa have been verified through thorough design and process reviews by the manufacturer, and are CE marked accordingly. The manufacturer of the device has conducted compatibility trials in line with the requirements of clause 9.1 of Annex 1 of the Medical Device Directive and ISO 80601, testing the AnaConDa in combination with ICU equipment provided by major manufacturers: ventilators, syringe pumps and gas monitors. The simultaneous use of these devices, however, creates a complex network, prone to human error. As with all medical devices, complaints and major incidents are logged, scrutinized and addressed by implementing design or instructional changes as the use of the device evolves and grows. Out of 260,000 uses 41 complaints have been made regarding the 100 mL AnaConDa. Data provided by the manufacturer, after correcting for escaped complaints by assuming that 50% more complaints are not detected, suggest a complaint rate of  0.016% for that device. This rate is considered low. Slightly more than half of those complaints (23) involved a cracking or leaking agent line. This was a result of an inadequate plastic weld at a weak point where the line fed into the evaporator, and was exacerbated by nurses’ tendency to fold IV lines to temporarily stop flow. The 50 mL AnaConDa has not been available long enough to generate similar complaints or data, however, the lessons learned from the 100 mL device were all applied to its development and manufacturing. The most cited clinical concerns surrounding the use of the devices are described and elaborated upon below.

### Titration

Concern over proper dosing and titration of isoflurane and sevoflurane via the AnaConDa may be due the limited familiarity among non-anesthetist staff with these agents, and the “off-label” nature of their use in the ICU [11, 15]. Meiser and Laubenthal suggested in 2005 that “the major reason why inhalation ICU sedation has not become more widely used, despite many favorable publications, is that, up until now, no commercially available ventilator fulfilled all the desired properties” [16]. Because of the low solubility of the agents, clearance is rapid. Furthermore, the concentration of the drug can be measured by a gas analyzer, and has a very direct relationship with the degree of sedation. This is a major advantage that volatile anaesthetics have over intravenous sedatives.

There have been several studies in which standard titration models have been tested. Belda et al. published a study in 2008 assessing the accuracy of a pharmacokinetic model for predicting dosing schemes with sevoflurane and the AnaConDa with some success. They were able to issue a table matching patient mass, tidal volume and infusion rates [17]. Tempia recommended that infusion rates above 15 mL/h and boluses must be used cautiously because they may result in excessive concentration peaks, requiring a dramatic consequential reduction in infusion rate. The use of boluses and high infusion rates makes drug titration unnecessarily more difficult and time consuming [3]—infusion rates should be adjusted gradually. The theoretically highest partial pressure a liquid agent could generate equals its vapor pressure at the prevailing temperature, i.e. 238 mmHg isoflurane and 157 mmHg sevoflurane or 31 and 20.7% of 1 atm at 20 °C (mmHg). While overdose is always a concern, in Meiser’s 2009 bench study he theorizes that the decrease in reflective efficiency at high concentrations might actually protect from overdose: at high doses, more drug washes through the filter [5]. Just like in the operating room, anaesthetic agent monitoring should be mandatory when using inhaled agents for sedation.

### Contraindication

The 100 mL AnaConDa is not intended for use in patients whose tidal volumes are less than 350 mL. The 50 mL device decreases this range to a minimum tidal volume of 200 mL. Patients requiring low flow, lung protective ventilation or with respiratory distress may contraindicate their use. With low flow protective ventilation, the small tidal volumes are insufficient to clear the device of CO_2_. In respiratory distress, low tidal volumes resulting in insufficient clearance of CO_2_ through the device will exacerbate the condition of patients who are already sensitive to hypercapnia. Both devices are contraindicated for use with high frequency ventilation and active humidification. The tidal volumes used in high frequency ventilation are similarly insufficient to clear CO_2_, and active humidification devices are positioned on the breathing circuit such that they will not fucntion properly.

Some problems have occurred in patients with excessive respiratory secretion, as reported by Bösel et al. [18]. In cases such as pneumonia the secretions can occlude the device, increasing resistance to breathing and decreasing reflection efficiency. Staff using the AnaConDa need to closely monitor secretions build-up in these patients and react accordingly.

Malignant hyperthermia is an absolute contraindication for the use of inhaled volatile anaesthetics, but is in no way related to the delivery systems.

### CO_2_ reflection

CO_2_ re-breathing has been reported to occur with the AnaConDa in certain situations, and this could lead to additional work of breathing [12] or higher than expected PaCO_2_. This issue was first raised by Sturesson et al. in 2009, who concluded that this may be relevant when tidal volumes are small, e.g. during lung protective ventilation [19, 20]. This CO_2_ reflection may cause the device to increase “apparent dead space” by approximately 23% to the volume of the 100 mL AnaConDa and require compensation for it through ventilator adjustments. In Sturessons’s investigation, there was a discrepancy between CO_2_ reflection in bench tests without humidity, and CO_2_ reflection with H_2_O vapor and anaesthetic agent present (which represents the situation when patients are being sedated using the AnaConDa), with CO_2_ reflection being higher in the “dry” bench study. It may be that there is competitive binding in the carbon felt between H_2_O and CO_2_, possibly accounting for the attenuation of CO_2_ reflection when moisture or anaesthetic is present. Their study might suggest that, if not immediately being used for sedation, the AnaConDa should be removed from the circuit and be replaced with a suitable HME filter to minimize risks of unnecessary rebreathing. Special attention may be required when even a small rise of PaCO_2_ might be detrimental to the patient, such as in patients with intracranial hypertension where there is increased intracranial pressure (ICP) [21]. Maintaining normocapnia when the AnaConDa is used may require a slight increase in ventilation, which can be maintained readily [20, 22]. If PaCO_2_ does increase [21, 23], it is up to the clinician to either accept the slightly elevated PaCO_2_ level, change the ventilator settings, or remove the AnaConDa.

### Environmental exposure

Environmental exposure and staff safety when working with inhaled volatiles using the AnaConDa in the ICU is another topic investigated in a number of clinician led trials and reports. The body of evidence demonstrates that room pollution is maintained well within acceptable limits if proper scavenging is used. Gonzalez Rodriguez demonstrated environmental safety using sevoflurane and *only* the AnaConDa, but recommended using either an active or passive scavenging system to ultimately ensure safety [24]. Using a charcoal filter placed at the ventilator exhaust port, Pickworth noted all levels were below 1 ppm [10]. In additional studies, the ambient concentrations of the drugs did not exceed the safe workplace exposure values [7–9, 11]. Sackey et al. corroborated this, and highlighted the importance of training and gaining confidence in handling the system to ensure pollution is minimal [6].

## Conclusion

Kong et al. [25] first proposed using inhaled volatiles for sedation in 1989. Sixteen years afterwards, however, Meiser suggested that the reason their use had not been more widely adopted at that point, in 2005, was that no commercially available ventilator fulfilled all the desired properties to deal with them in the ICU [16]. In the early practise of using volatiles as sedatives, operating room anaesthesia ventilators and gas vaporizers were deployed in the ICU along with their attendant expert clinicians, both at impractical expense. Compounding that impracticality is the inability of OR ventilators to match the therapeutic breathing modes and performance of current ICU ventilators, crucial to contemporary respiratory therapy. In comparison with operating rooms, fewer room air-changes per hour in most ICU facilities increase the risk of patients and practitioners being exposed to leaked or exhausted isoflurane or sevoflurane. Perhaps in their 2005 comment Meiser and company meant that no sound delivery system existed that would allow the use of the drugs in the ICU without extraordinary expense or technical difficulty while maintaining patient and practitioner safety.

The AnaConDa seems to fill this role, not simply by delivering the drugs but by doing so in concert with equipment common to almost every ICU. Inserted into the ventilator circuit, it uses existing ventilator machines with virtually no physical modifications needed. Ubiquitous syringe pumps deliver a measured dose of isoflurane or sevoflurane to the device. By providing a real time indication of drug concentration, conventional gas monitors are used to titrate the drugs to the desired sedation level. The AnaConDa reduces drug use and sedation costs by reflecting 90% of it back to the patient while functioning simultaneously as a humidity and moisture exchanger and provides fresh gas flow to the patient. Furthermore, the device is single-use, is of simple construction and is disposable, making any additional post-use maintenance to the ventilators it is used with unnecessary. While the AnaConDa is relatively inexpensive, there is a currently a lack of real, long term data exploring the economics of volatile use; including the device, costs of monitoring, the value of predictable waking and shorter weaning, length of patient ICU and hospital stays, and any additional therapeutic benefits that the volatile drugs may offer.

Should the use of inhaled volatile anaesthetics as ICU sedatives expand, the AnaConDa device seems uniquely poised to provide a simple, safe and cost-effective means of delivering isoflurane and sevoflurane.
